# Investigating the role of costimulatory domains in enhancing CAR-T cell persistence in TNBC

**DOI:** 10.1038/s41417-026-01073-0

**Published:** 2026-08-21

**Authors:** Hany E. Marei, Giacomo Pozzoli, Carlo Cenciarelli

**Affiliations:** 1 Department of Cytology and Histology, Faculty of Veterinary Medicine, Mansoura University, Mansoura, Egypt; 2 Section of Pharmacology, Department of Translational Medicine and Surgery, Università Cattolica del Sacro Cuore-Fondazione Policlinico Universitario “A. Gemelli” IRCCS, Rome, Italy; 3 Institute of Translational Pharmacology-CNR, Rome, Italy

**Keywords:** Breast cancer, Immunosuppression

## Abstract

This work summarizes how costimulatory domains can mediate the persistence, function, and therapeutic potential of CAR-T cells in triple-negative breast cancer (TNBC), a highly aggressive malignancy with limited treatment options and an immunosuppressive tumor microenvironment. Although CAR-T cell therapy has produced remarkable results in hematologic malignancies, its translation to TNBC remains limited by poor T cell persistence, metabolic dysfunction, and rapid exhaustion. Current evidence identifies canonical costimulatory domains (CD28 and 4-1BB) as having differential effects on intracellular signaling, metabolic programming, and T cell differentiation; CD28-based CAR-T cells promote rapid activation and glycolytic metabolism but are commonly associated with terminal differentiation and decreased durability, whereas 4-1BB signaling supports mitochondrial fitness, oxidative phosphorylation, and the development of memory-like T cells that support longer-lasting persistence. The functional differences in CAR-T cells are context-dependent and influenced by antigen density, hypoxia, and other immunosuppressive signals within the TNBC tumor microenvironment. Newer costimulatory domains, such as HVEM and TNFRSF9, have added an additional layer of complexity to T cell signaling by modulating activating and inhibitory pathways, and their incorporation into CAR designs offers new ways to optimally regulate T cell responses; however, their functions are not fully defined in TNBC models. Additionally, dual costimulation strategies and combination therapies (metabolic reprogramming of T cells, epigenetic modulation, and immune checkpoint blockade) have shown promise in preclinical studies, but their translational value remains to be validated. An important message arising from this review is that while persistence may be improved through enhanced costimulatory signaling, the ideal performance of CAR T therapy occurs with the establishment of an ideal balance of stimulating signals that promote the sustained function of effector T cells while inhibiting the exhaustion of T cells and avoiding the deleterious effects of tonic activation. Furthermore, it is critical to evaluate data from TNBC models compared to other solid tumors to inform rational CAR T design strategies in TNBC.

## Introduction

Defined by the absence of estrogen receptor (ER), progesterone receptor (PR), and human epidermal growth factor receptor 2 (HER2) expression, triple-negative breast cancer (TNBC) accounts for approximately 15–25% of all breast cancer cases [1, 2]. The lack of actionable molecular targets limits therapeutic options and contributes to high recurrence rates, metastatic potential, and poor overall prognosis [2, 3]. Although advances in immune checkpoint inhibitors (ICIs) and chemotherapy have improved outcomes for some patients, a substantial proportion still fail to achieve durable responses, underscoring the urgent need for more effective treatment strategies [3, 4].

The main methods of treating triple-negative breast cancer (TNBC) are chemotherapy, surgery, and radiation therapy. Unfortunately, these methods often have high rates of return to the cancer (relapses) and limited ability to control the cancer over the long term in advanced/metastatic disease [5, 6]. Some newer therapies, such as immune checkpoint inhibitors (ICIs), such as anti-PD-1/PD-L1, have shown that a small group of patients with TNBC can have improvement in outcomes; however, only modestly effective in producing longer-lasting responses and responses are often not maintained long-term after stopping the treatment [3, 4]. By comparison, the antitumor effect of CAR-T cell therapy has the ability to be sustained by the target specificity of the antigens and the ability to expand in vivo via different strategies, which has led to long-lasting remission in hematological malignancies [7]. On the other hand, despite having shown some clinical outcomes from the use of CAR-T cell therapy in TNBC and other solid tumors, there have been limited examples of similar successes in solid tumors compared to the successes seen with blood cancers. This is mainly due to the difficulty of persistence, lack of tumor infiltration and degree of immunosuppression associated with these solid tumors [8, 9].

TNBC represents a particularly compelling target for CAR-T cell therapy due to both its unmet clinical need and its immunobiological features. Unlike hormone receptor–positive or HER2-amplified breast cancers, TNBC lacks well-defined molecular targets, limiting the effectiveness of targeted therapies and contributing to poorer clinical outcomes [1, 2]. In addition, TNBC tumors often exhibit higher levels of tumor-infiltrating lymphocytes (TILs) and increased expression of immune-related genes, suggesting a degree of intrinsic immunogenicity that may be leveraged by adoptive cell therapies [4, 10]. However, current immunotherapeutic approaches, including immune checkpoint inhibitors, have demonstrated limited and variable efficacy, highlighting the need for alternative strategies capable of inducing more robust and durable antitumor responses [3, 4]. In this context, CAR-T cell therapy offers a promising approach by enabling antigen-specific targeting and sustained immune activation independent of major histocompatibility complex (MHC) restriction.

While CAR-T cell therapy has optimistic potential in TNBC, there are numerous tumor-intrinsic and microenvironmental obstacles to the use of this therapy, including heterogeneity of the target surface antigen(s), mechanical barriers to T cell penetration of the tumor, and an extremely immunosuppressive tumor microenvironment consisting of regulatory T cells (Tregs), myeloid-derived suppressor cells (MDSCs), and inhibitory cytokines [8, 11]. In fact, one of the primary limitations to achieving long-term clinical responses in solid tumors with CAR-T cell therapy, regardless of tumor type, is insufficient persistence of CAR-T cells in the tumor [8, 9]. This characteristic underscores the importance of optimizing CAR design, particularly the selection of costimulatory domains, as they play an essential role in regulating T cell activation, metabolic fitness, and resistance to exhaustion. Thus, understanding how costimulatory domains mediate CAR-T cell persistence in TNBC is essential for enhancing the clinical utility of CAR-T cell therapy and facilitating its clinical translation.

Chimeric antigen receptor (CAR) T-cell therapy has demonstrated remarkable success in hematologic malignancies by enabling T cells to recognize and eliminate tumor cells independently of major histocompatibility complex (MHC) presentation [7, 12]. However, translating this success to solid tumors such as TNBC remains challenging [8, 13].

Triple-negative breast cancer (TNBC) has the characteristics of having limited treatment options once there has been the development of resistance to first-line therapy and of early dissemination [5, 6]. Current studies to determine whether CAR-T cell therapies will be effective at controlling metastatic TNBC in the clinical setting are still being conducted [8]. Some pre-clinical studies have shown that CAR-T cells are able to reduce tumor burden or delay progression of tumors in solid tumor models; however, these results have yet to be translated into consistent, durable disease control in patients [8, 14, 15]. Therefore, CAR-T therapy represents an interesting approach and will continue to be studied to determine its usefulness in controlling metastatic TNBC.

When assessing CAR-T cell treatments, and how well they work, how patients feel and the adverse effects from treatment need to be taken into consideration. CAR-T treatments have been shown to produce long-term responses in some cases; however, they also produce very serious adverse effects, such as Cytokine Release Syndrome and Immune Effector Cell Associated Neurological Toxicity. These conditions may affect how individuals feel and require a significant amount of medical care [16, 17]. CAR-T treatments require a complex manufacturing process and hospital admission. The complexity of these treatments may limit accessibility and overall patient experience. Currently, there is minimal data comparing quality of life outcomes between CAR-T and standard TNBC treatment. Further clinical trial evaluation is needed to assess how well the CAR-T therapy works and how well CAR-T patients do compared to patients receiving standard TNBC treatment.

In TNBC, CAR-T cell efficacy is limited by several factors, including antigen heterogeneity, an immunosuppressive tumor microenvironment (TME), and insufficient CAR-T cell persistence [8, 9, 13]. Among these, persistence has emerged as a critical determinant of therapeutic success, as inadequate durability of CAR-T cells contributes significantly to treatment failure [9, 12].

Compared to first-generation CARs, second-generation CAR-T cells incorporate costimulatory domains such as CD28 or 4-1BB, which enhance T-cell activation, proliferation, and survival [18, 19]. These domains differentially regulate intracellular signaling pathways and metabolic programming. CD28-based CAR-T cells promote rapid activation and glycolytic metabolism but are often associated with terminal differentiation and reduced persistence [19–21]. In contrast, 4-1BB signaling supports mitochondrial biogenesis, oxidative phosphorylation, and the development of memory-like T cells that sustain long-term persistence [19, 22]. More advanced CAR designs, including third- and fourth-generation constructs, incorporate multiple or alternative costimulatory domains—such as ICOS, OX40, TNFRSF9, and HVEM—to further modulate CAR-T cell function [18, 23]. However, the optimal combination and configuration of these domains in the context of TNBC remain incompletely understood.

Metabolic fitness is a key determinant of CAR-T cell persistence, particularly within the nutrient-deprived and hypoxic TNBC tumor microenvironment [21, 24]. Immunosuppressive factors, including myeloid-derived suppressor cells (MDSCs), regulatory T cells (Tregs), and inhibitory cytokines such as TGF-β and IL-10, further impair CAR-T cell function [11, 13, 24]. In addition, epigenetic regulation has emerged as an important contributor to CAR-T cell durability, although the extent to which costimulatory domains influence epigenetic programming remains poorly defined [25]. The lack of TNBC-specific preclinical models also limits the ability to evaluate these mechanisms in a disease-relevant context [8]. Developing robust systems, including three-dimensional tumor cultures and patient-derived xenograft (PDX) models, will be essential for validating strategies aimed at improving CAR-T cell persistence.

This review investigates how costimulatory domains may enhance TNBC CAR-T cell persistence and efficacy. We aim to identify effective strategies to optimize CAR-T therapy by comparing conventional domains such as CD28 and 4-1BB with emerging alternatives including TNFRSF9 and HVEM. Moreover, we examine how costimulatory signaling influences metabolic programming, resistance to immunosuppressive signals, and epigenetic regulation. In particular, this review focuses on the mechanistic effects of individual costimulatory domains on CAR-T cell signaling, metabolic programming, and persistence, with emphasis on evidence derived from TNBC models where available. Synthesizing current research, we propose rational engineering approaches—including dual costimulation, metabolic adaptation, and epigenetic modulation—to address current limitations and improve CAR-T therapy for TNBC in clinical practice.

Recent literature has concentrated on CAR-T therapy in solid tumors and the role of costimulatory signals in T cell activity [26–29]. Previous work has summarized challenges in implementing CAR-T therapy in solid tumors, including difficulty with tumor infiltration, antigen heterogeneity, and immune system suppression; engineering approaches to enhance CAR-T cell activity have also been described [27, 29]. Some reviews have described the mechanisms of action of costimulatory signals, including CD28 and 4-1BB, in determining T cell activation and metabolic status [19, 20]. However, previous reviews have focused on general CAR-T cell biology or the broad application of CAR-T cells to solid tumors, rather than providing specific information on the challenges of treating TNBC or discussing the mechanistic relationship between costimulatory signals and the persistence of CAR-T cell therapy in this disease. In this review, we examine the regulation of CAR-T cell persistence using a combined approach that integrates the different signals received by CAR-T cells and the metabolic differences among them, as well as the unique characteristics of the tumor microenvironment associated with TNBC.

Several recent reviews have examined CAR-T cell therapy in solid tumors and the role of costimulatory signaling in T-cell function [26–29]. For example, prior studies have summarized the challenges of CAR-T therapy in solid malignancies, including tumor infiltration, antigen heterogeneity, and immune suppression, as well as strategies to enhance CAR-T cell activity through engineering approaches [27, 29]. Other reviews have discussed the molecular mechanisms of costimulatory domains, particularly CD28 and 4-1BB, in shaping T-cell activation and metabolism [19, 20]. However, these studies have largely focused on general CAR-T cell biology or broad solid tumor applications, with limited emphasis on the specific challenges of triple-negative breast cancer (TNBC) or the mechanistic relationship between costimulatory signaling and CAR-T cell persistence in this context. In contrast, the present review integrates signaling, metabolic, and tumor microenvironment–specific factors to provide a focused analysis of how costimulatory domains regulate CAR-T cell persistence in TNBC.

This review links costimulatory domain signaling with persistence outcomes for CAR-T cells in the TNBC tumor microenvironment to provide a unique, targeted design framework for the creation of CAR-T cells. We also describe new costimulatory domains (e.g., HVEM, TNFRSF9) and specifically discuss their potential contribution, in addition to established domains, to long-term CAR-T cell function. The present work is distinct from former reviews in that it provides a narrow focus and identifies numerous opportunities for potential future investigation.

Distinguishing results from investigations using TNBC-specific models from those using hematologic malignancy or other solid tumor models is an important consideration. CAR-T cell studies of leukemia and lymphoma have thus far provided the basis for understanding the role of costimulatory signaling and persistence; however, the characteristics of the TNBC’s tumor microenvironment lead to unique issues: antigen heterogeneity, anoxic conditions, and immunosuppressive cellular networks [5, 11]. Likewise, data from other solid tumor types can yield mechanistic information about the metabolism and persistence of CAR-T cells, but their relevance to TNBC must be interpreted carefully because of differences such as stroma composition, antigen density, and the ability to evade immune destruction [8, 11]. In this article, we aim to identify TNBC-specific evidence separately from that of other tumor types to accurately characterize the role of the costimulatory domain in CAR-T cell persistence in TNBC.

The difficulties listed above illustrate that further rational development of CAR-T cell engineering methods is necessary to create newer, more effective treatments available for patients with TNBC.

## Costimulatory domains and different generations of Car-T cells

**Fig. 1:**
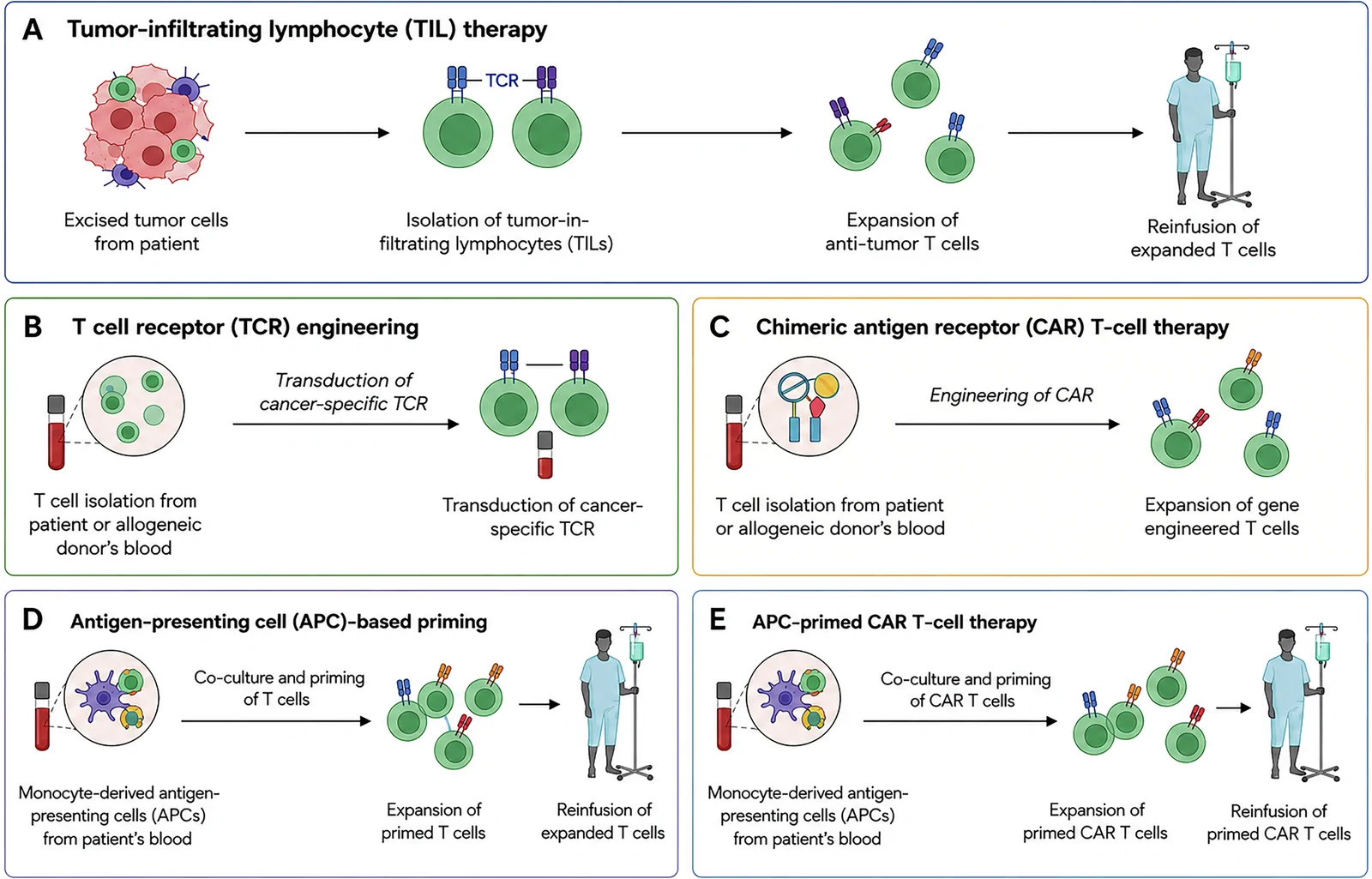
Schematic representation of the main strategies for adoptive T-cell therapy to treat tumors. **A** Tumor-infi ltrating lymphocyte (TIL) therapy. Tumor tissue is obtained from the patient, and TILs are isolated from the excised tumor, expanded ex vivo, and subsequently reinfused into the patient. **B** T-cell receptor (TCR) engineering. T cells isolated from the patient or an allogeneic donor are genetically modifi ed ex vivo to express tumor-specifi c TCRs and expanded for therapeutic use. **C** Chimeric antigen receptor (CAR) T-cell therapy. T cell sisolated from the patient or an allogeneic donor are genetically engineered to express CARs that recognize tumor-associated antigens, followed by ex vivo expansion and reinfusion. **D** Antigen-presenting cell (APC)-based priming. Monocyte-derived APCs are generated from the patient's blood and co-cultured with T cells to promote tumor-antigen-specifi c activation and expansion before reinfusion. (E) APC-primed CAR T-cell therapy. Monocyte-derived APCs are co-cultured with CAR T cells to enhance their activation and expansion prior to reinfusion into the patient. Collectively, these approaches rely on ex vivo activation and/or genetic modifi cation, expansion, and subsequent reintroduction of therapeutic T cells. They diff er principally in the mechanisms by which tumor antigens are recognized and, for TCR- and APC-dependent approaches, in their reliance on major histocompatibility complex (MHC)-mediated antigen presentation. The figure was created with BioRender.com [7, 8, 30, 78].

While multiple T cell–based immunotherapies exist, this review focuses specifically on CAR-T cell engineering, with emphasis on how costimulatory domains regulate persistence in triple-negative breast cancer (TNBC). Other adoptive T-cell approaches, including tumor-infiltrating lymphocyte (TIL) therapy and T cell receptor (TCR) engineering, have shown therapeutic potential; however, this review centers on CAR-T cells due to their capacity to recognize tumor antigens independently of major histocompatibility complex (MHC) presentation and their adaptability through genetic engineering [15, 30, 31] (Fig. 1).

**Fig. 2:**
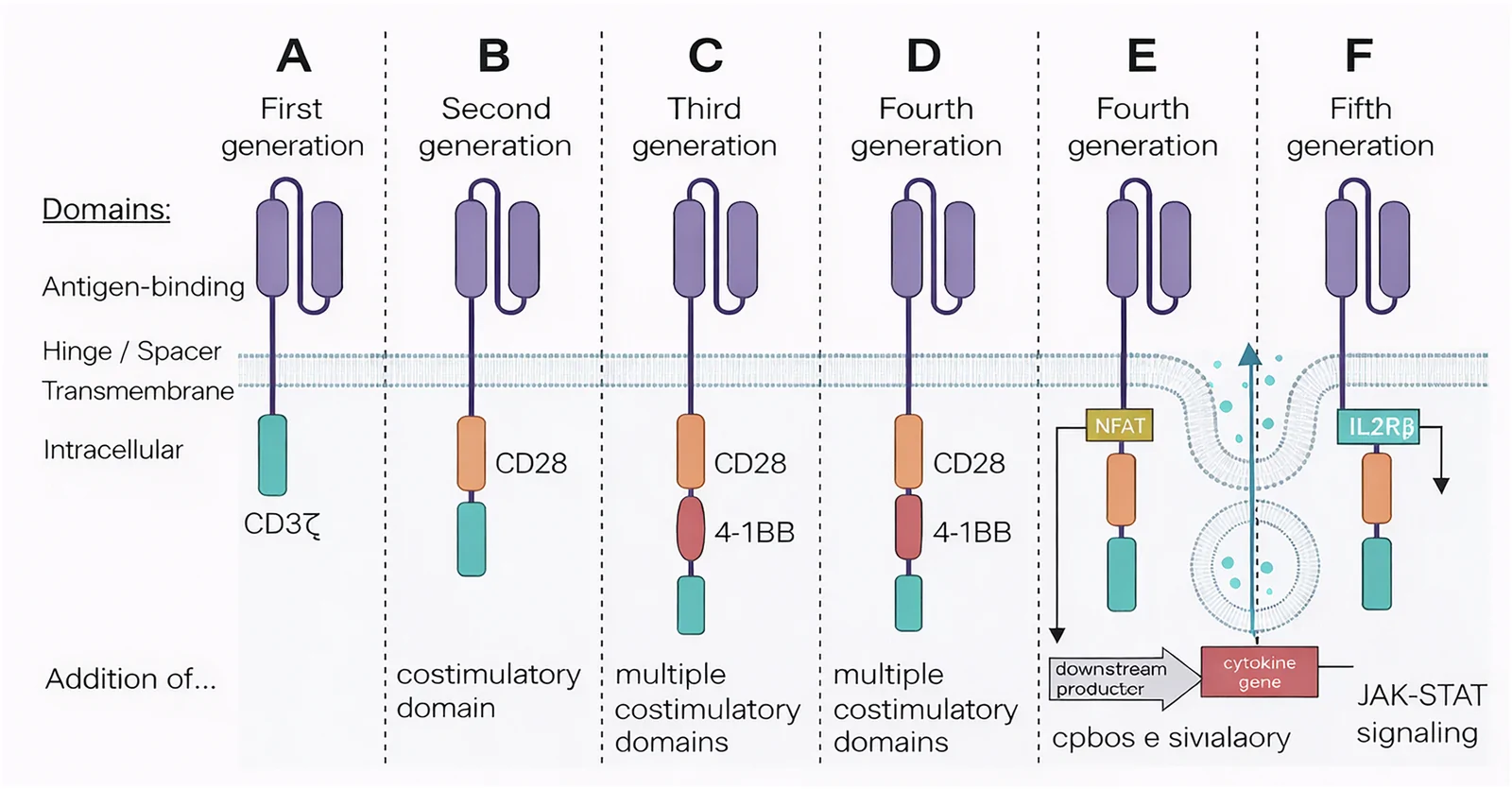
Schematic showing the evolution of CAR-T cells over multiple generations and an overview of the co-stimulator domain. **A** The first generation of CARs has an antigen-binding extracellular domain (generally a single-chain variable fragment (scFv)) to bind a specific antigen, a hinge region, and a transmembrane region from the scFv domain to link to the intracellular portion of the CAR containing a CD3ζ signaling domain to activate T cells in the absence of costimulation. **B** The second generation of CARs has one costimulation domain (for example, either CD28 or 4-1BB) added to the CD3ζ signaling domain to increase T cell activation and proliferation and enhance T cell persistence. **C** The third generation of CARs has two costimulatory domains (typically CD28 and 4-1BB) to increase T cell signaling and enhance T cell function. **D** The fourth generation of CARs, called a TRUCK, can be designed to induce the production of cytokines or additional co-stimulatory ligands through methods that utilize NFAT transcription, allowing CAR-T cells to alter the tumor microenvironment. **E** Enhanced fourth-generation CAR constructs have further modified T cell signaling through the addition of regulatory elements, resulting in increased production of cytokines from T cells and increased local immune activation. **F** The fifth generation of CARs will contain intracellular signaling domains that are derived from cytokine receptors (such as IL-2Rβ), allowing the CAR-T cells to activate JAK-STAT signaling pathways and achieve sustained proliferation, survival, and long-term persistence. This diagram summarizes how the addition of co-stimulator and cytokine signaling domains throughout the evolution of CAR-T cells will affect T cell signaling, metabolic programming, and long-term persistence. The illustration was created utilizing previously published work that describes CAR T cell design and function, and was created using BioRender.com [18, 19, 24].

CAR-T cell therapy has evolved through several generations of design, with second-generation CARs introducing costimulatory domains such as CD28 or 4-1BB, which significantly enhance T-cell activation, proliferation, and survival compared to first-generation constructs that rely solely on CD3ζ signaling [18, 19, 23]. Subsequent CAR designs have incorporated multiple or alternative costimulatory domains to further refine T-cell function and persistence [5, 7] (Fig. 2).

In the context of TNBC, the selection of costimulatory domains is a key determinant of CAR-T cell persistence, as these domains regulate intracellular signaling pathways that influence metabolic fitness, differentiation state, and resistance to exhaustion within the tumor microenvironment [8, 11, 23, 32].

### Integrated signaling pathways governing CAR-T cell persistence

**Fig. 3:**
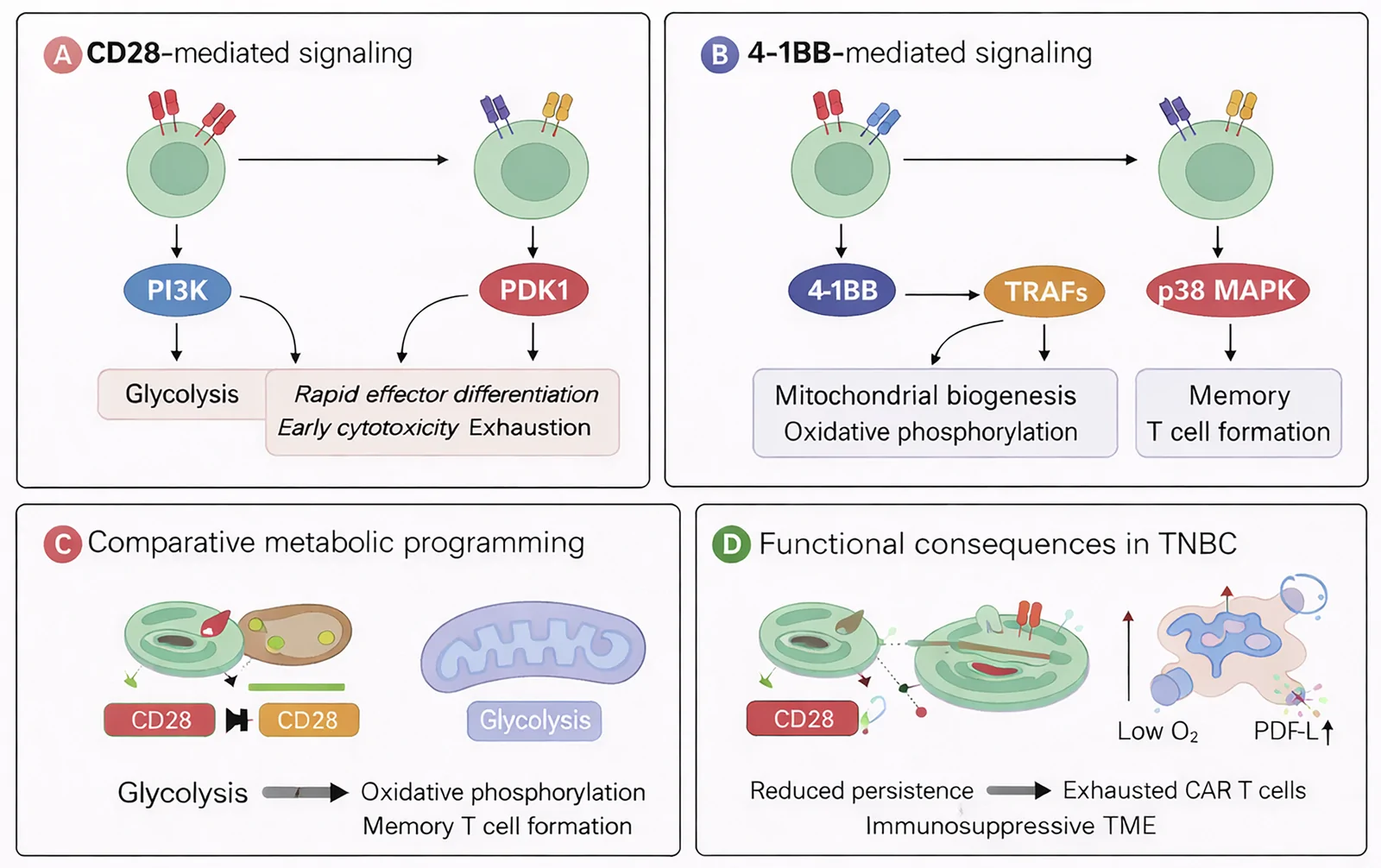
Costimulatory domain–dependent signaling pathways regulating CAR-T cell persistence. **A** CD28-mediated signaling activates the PI3K–AKT–mTOR pathway, promoting glycolysis, rapid effector differentiation, and early cytotoxicity but contributing to exhaustion under reduced persistence. **B** 4-1BB–mediated signaling activates TRAF-dependent pathways, including NF-κB and p38 MAPK, supporting mitochondrial biogenesis, oxidative phosphorylation, long-term persistence, and memory T-cell formation. **C** Comparative metabolic programming: CD28 favors glycolytic metabolism, whereas 4-1BB promotes oxidative metabolism and long-term persistence. **D** Functional consequences in TNBC: differential signaling affects CAR-T cell persistence, exhaustion, and resistance to immunosuppressive conditions in the tumor microenvironment. This schematic summarizes key intracellular signaling pathways activated by major costimulatory domains and their impact on CAR-T cell persistence, metabolism, and functional differentiation. The framework is derived from studies investigating CD28- and 4-1BB-mediated signaling, tonic signaling, and mechanisms regulating CAR-T cell exhaustion and survival [9, 19, 20, 22, 35]. CD28 signaling is associated with PI3K–AKT–mTOR activation and glycolytic metabolism, whereas 4-1BB signaling promotes mitochondrial biogenesis and oxidative phosphorylation through NF-κB–dependent pathways. These signaling differences are critical determinants of CAR-T cell persistence, particularly in the metabolically restrictive tumor microenvironment of TNBC. The figure was developed using BioRender.com.

In TNBC, costimulatory domains modulate CAR-T cell persistence primarily through differential activation of signaling pathways that control metabolism, survival, and differentiation under immunosuppressive conditions. Costimulatory domains function independently of their structural classification to activate intracellular signaling cascades that ultimately determine CAR-T cell fate, particularly within the hostile tumor microenvironment characteristic of solid tumors such as TNBC (Fig. 3) [19, 24].

CD28-based CAR-T cells predominantly activate the PI3K–AKT–mTOR signaling axis, which promotes rapid effector differentiation, glycolytic metabolism, and immediate cytotoxic function [19, 20, 23]. While this signaling profile enhances early tumor clearance, sustained mTOR activation has been associated with terminal differentiation and increased susceptibility to exhaustion, particularly under nutrient-deprived and hypoxic conditions typical of the TNBC tumor microenvironment [20, 21]. In contrast, 4-1BB–based CAR-T cells preferentially engage TRAF-mediated signaling pathways, including non-canonical NF-κB and p38 MAPK, which support mitochondrial biogenesis, oxidative phosphorylation, and the development of central memory-like T-cell phenotypes [19, 22]. Long-term persistence and continued antitumor activity are associated with these biopsy-transcriptional programs, which were mostly described in non-TNBC solid tumor or hematologic models, in solid tumours, including TNBC [9, 22].

**Fig. 4:**
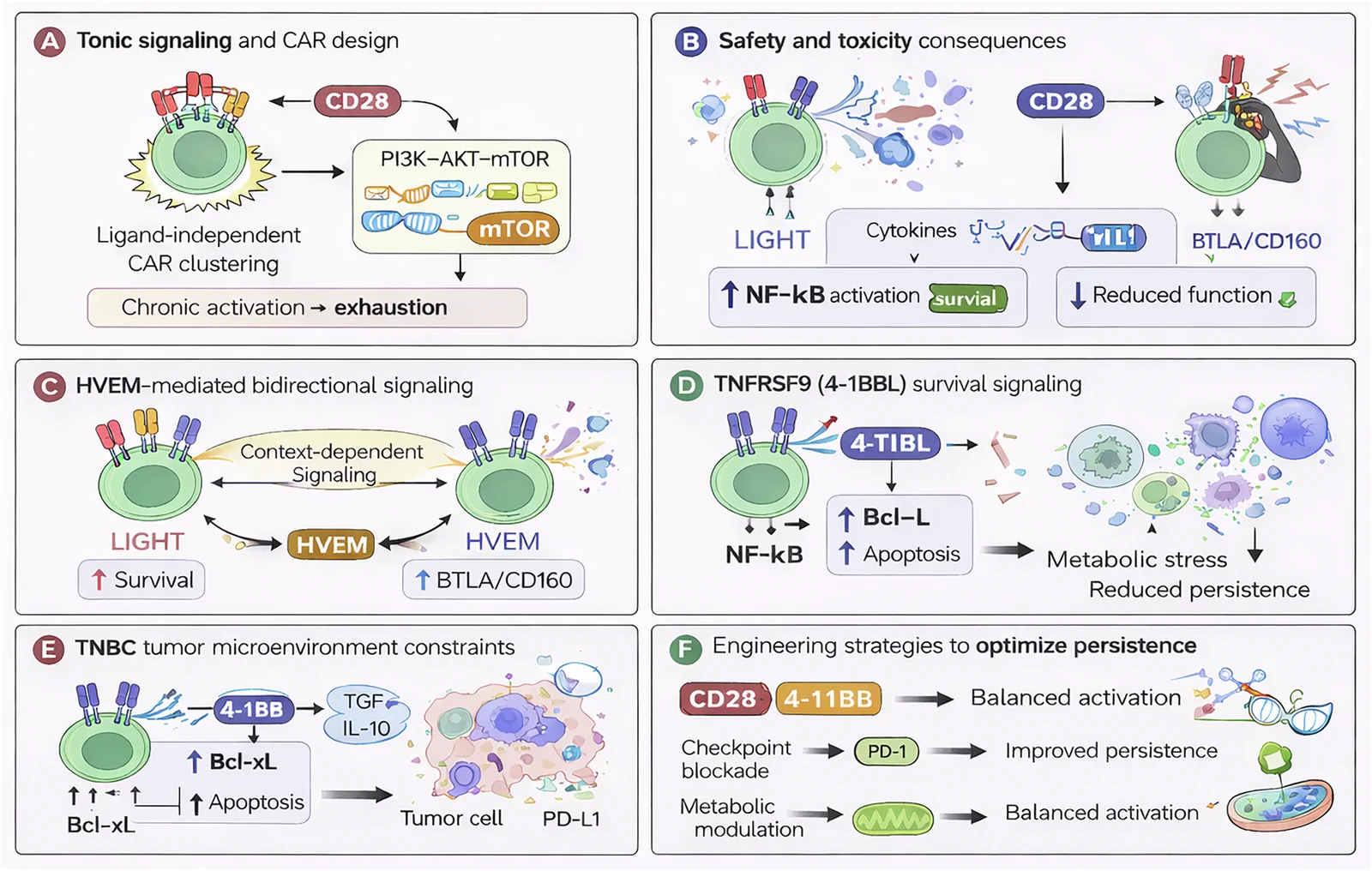
Additional mechanisms regulating CAR-T cell persistence, safety, and therapeutic efficacy in TNBC. **A** Tonic signaling of the CAR design is a result of receptor clustering due to the high degree of affinity of the scFv and will happen before there is any interaction with a ligand, and as a result of being activated by a ligand, the CAR activates the PI3K-AKT-mTOR pathway, resulting in T-cell exhaustion. **B** If there are high levels of costimulation, then there will be a high level of cytokine release, thus putting the patient at increased risk of developing CRS and ICANS. **C** Although HVEM’s signaling pathway is capable of a bidirectional regulatory effect, an interaction with LIGHT leads to the induction of NF-kB (in both T cells and LIGHT-responsive cells), and these signals will lead to increased survival of T cells through activation of the NF-kB pathway (i.e., Bcl-xL). Conversely, if the T cells express either BTLA or CD160, then those receptors will provide T-cell inhibition signals that are dependent on the strength of activation and the time since activation. **D** T-cells are activated through the engagement of 4-1BBL (TNFRSF9) and the resulting engagement of the NF-kB pathway will provide for prolonged CAR T-cell persistence. **E** Factors in the TNBC tumoral microenvironment that influence CAR T-cell functionality include a hypoxic environment, immunosuppressive cytokines (TGF-beta and/or IL-10), regulatory immune cells, and immune checkpoint signaling, leading to T-cell metabolic dysfunction and exhaustion. **F** Strategies aimed at optimizing CAR-T persistence include, but are not limited to, balancing activation and persistence via dual co-stimulation, checkpoint blockade, and metabolic reprogramming/epigenetic modifications. These diagrams illustrate results or mechanisms arising from scientific investigations of CAR-T-cell signaling (i.e., tonic activation of CAR-T cells), CAR-T cellular interaction with the tumor microenvironment, and next-generation engineering and were created using BioRender.com [19, 20, 22, 35, 67].

Emerging costimulatory domains, such as HVEM and TNFRSF9, introduce additional layers of signaling complexity by modulating the balance between activating and inhibitory pathways within the tumor microenvironment (Fig. 4). HVEM functions as a bidirectional signaling receptor, capable of promoting NF-κB activation through interaction with LIGHT, while also mediating inhibitory signaling via BTLA or CD160, thereby influencing T-cell activation in a context-dependent manner [33, 34]. Similarly, TNFRSF9 (4-1BBL) signaling enhances CAR-T cell survival and persistence through activation of anti-apoptotic pathways, including upregulation of Bcl-xL, and supports mitochondrial fitness [22]. These domains may contribute to improved CAR-T cell durability by promoting resistance to apoptosis and maintaining functional capacity during prolonged antigen exposure. However, their precise effects in TNBC remain incompletely defined.

Collectively, these findings suggest that different costimulatory domains regulate CAR-T cell persistence by shaping distinct signaling networks that control metabolic programming, differentiation state, and resistance to exhaustion. In the context of TNBC, where the tumor microenvironment imposes significant metabolic and immunosuppressive constraints, the selection and optimization of costimulatory domains represent critical determinants of therapeutic efficacy.

A significant proportion of these mechanistic insights are derived from hematologic malignancies or non-TNBC solid tumor models. While these findings provide a valuable framework, their direct applicability to TNBC remains to be fully validated and should be interpreted with caution.

The majority of mechanistic models detailing the costimulatory signaling pathway (e.g., the PI3K–AKT–mTOR pathway or that of NF-κB) have been developed by studying hematologic malignancies or using model systems based on non-TNBC solid tumors [19, 20]. Additionally, although these studies have provided essential information regarding CAR-T cell biology, they do not always translate well to TNBC, due to unique characteristics associated with TNBC cells and their related immune suppression, which may ultimately impact the signaling dynamics for CAR-T cells and persistence [1, 11]. Thus, in instances where minimal or no TNBC-specific data are available, conclusions should be viewed as hypothesis-generating rather than conclusive.

In particular, improved CAR-T cell persistence does not uniformly accompany increased costimulatory signaling. Tonic signaling is constituted independently of ligand activation may occur as a result of features of the CAR design, such as scFvs having high affinities, spontaneous clustering of the receptors, and strong costimulatory domains [19, 20]. Tonic signaling via CD28-containing CAR constructs is associated with enhanced activation of the PI3K-AKT-mTOR signaling cascade, resulting in early T-cell exhaustion and impaired longevity [19, 35]. In contrast, tonic signaling via 4-1BB-containing CAR constructs is associated with improved mitochondrial function, memory development, and a reduced likelihood of dysfunction due to persistent exposure to antigen [19, 22]. In TNBC, where CAR-T cells are continuously exposed to antigen stimulation and an immunosuppressive environment, excessive tonic signaling could increase metabolic stress and impair CAR-T cell function.

Excessive co-stimulation can lead to serious safety concerns and negatively affect the persistence of CAR-T cells (see Fig. 4). The degree of stimulation from a strong co-stimulatory domain has also been shown to be associated with higher levels of cytokine production; increased activation may place a patient at greater risk of developing CRS or other inflammatory toxicities [16, 31]. In addition, persistent activation can contribute to activation-induced cell death and immune system dysregulation due to prolonged signaling, thereby limiting the durability of CAR-T cells as a therapy [7, 20]. Specific design parameters of CAR-T cells, such as co-stimulatory domain choice, scFv affinity, hinge/transmembrane design, and expression level, can greatly influence both the tonic signaling and the safety profile of CAR-T cell therapy [24, 35]. Therefore, the results of this study suggest that optimizing persistence of CAR-T cells in TNBC requires adequate calibration of co-stimulation magnitude to achieve a suitable compromise among efficacy, long-term function, and patient safety.

Regarding TNBC, it has been determined that excessive co-stimulation signaling could lead to an accelerated rate of CAR T cell exhaustion and risk of toxicity and that it would be more important to design signals to be balanced rather than maximally activated.

### CD28 domain

Within TNBC-relevant models, CD28 costimulation is associated with rapid effector differentiation and strong initial cytotoxicity but limited long-term persistence due to increased susceptibility to exhaustion in the immunosuppressive tumor microenvironment [9, 19, 20]. CD28 provides a critical costimulatory signal through interaction with CD80/CD86 ligands, leading to enhanced T-cell activation, proliferation, cytokine production, and survival [36]. In CAR-T cells, incorporation of the CD28 intracellular domain activates downstream signaling pathways, particularly PI3K–AKT–mTOR, which promote glycolytic metabolism and effector T-cell differentiation [20, 23]. These signaling characteristics enable rapid tumor cell killing but are also associated with terminal differentiation and reduced persistence under chronic antigen exposure [20, 21]. This means that in triple-negative breast cancer, the CD28 costimulation pathway increases effector functions in the early stages through the traditional glycolytic pathway that is primarily controlled by the PI3K-AKT-mTOR pathway, but it also limits the persistence of CAR-T cells due to an increased rate of differentiation and an increased risk of experiencing exhaustion once located within the tumor microenvironment.

Studies in TNBC and other solid tumor models are investigating the efficacy of CD28-based CAR-T cells targeting tumor-associated antigens (TAAs), including HER2, MUC1, and EGFR, with the aim of improving tumor specificity and therapeutic outcomes [13, 37]. CD28-based CAR-T cells promote antitumor activity through increased production of cytokines such as IL-2 and IFN-γ, which support T-cell expansion and effector function [23]. However, the TNBC tumor microenvironment, characterized by the presence of regulatory T cells (Tregs), myeloid-derived suppressor cells (MDSCs), and inhibitory cytokines such as IL-10 and TGF-β, imposes significant constraints on CAR-T cell activity and persistence [11, 13]. While CD28 signaling enhances early activation and pro-inflammatory responses, these same features may accelerate exhaustion in the context of sustained antigen stimulation within solid tumors [20, 21].

In preclinical trials involving solid tumors without triple-negative breast cancer (TNBC), CD28-based CAR-T cells showed superior early tumor clearance compared with 4-1BB CARs. This was especially evident in models of tumors designed to express both CD19 and EGFR [20, 22]. However, these responses are often not durable, as CD28-driven CAR-T cells tend to decline more rapidly over time [22, 38]. In TNBC-specific models, combination strategies have been explored to overcome these limitations. For example, CAR-T cells targeting carcinoembryonic antigen (CEA) with CD28ζ signaling have shown enhanced cytotoxicity in vitro, and when combined with image-guided radiation therapy (IGRT), demonstrated improved tumor control and CAR-T cell infiltration in orthotopic TNBC mouse models [39]. These findings suggest that augmenting CD28-based CAR-T cells with complementary therapeutic approaches may improve their persistence and efficacy.

Additional strategies to enhance CD28-based CAR-T cell performance include combining CAR-T therapy with immune checkpoint inhibitors, engineering CAR-T cells to secrete pro-inflammatory cytokines, and modifying chemokine receptor expression to improve tumor infiltration [8, 11]. Advances in gene-editing technologies, such as CRISPR/Cas9, have also enabled targeted modification of inhibitory pathways and immune checkpoint regulators to improve CAR-T cell persistence [40]. Furthermore, optimizing CAR design parameters—including receptor affinity and costimulatory domain combinations—has been shown to influence functional outcomes and resistance to immunosuppression [41].

Despite these advances, a substantial portion of the mechanistic understanding of CD28 signaling is derived from hematologic malignancies or non-TNBC solid tumor models. Therefore, the extent to which these findings translate to TNBC remains under active investigation. In the context of TNBC, these findings suggest that CD28-driven signaling enhances early tumor control but may compromise persistence unless combined with strategies that mitigate exhaustion [20, 35].

Studies on CD28-mediated signaling to understand why CAR-T cells persist have mostly examined blood cancers; however, there are also studies showing both similarities and differences between TNBC and blood cancers. An example of this is that CD28 signaling promotes rapid differentiation into terminal effector cells across multiple systems, but at the same time does not promote CD28-mediated CAR-T cell persistence like it does in TNBC due to increased metabolic challenge and immunosuppressive signaling in the TNBC tumor microenvironment [1, 11, 19, 20]. Therefore, care should be taken when using CD28-based CAR-T cell strategies in TNBC due to the lack of concordance with other non-TNBC settings.

### 4-1BB (CD137) costimulatory domain

In TNBC-associated models, 4-1BB costimulation promotes mitochondrial biogenesis and oxidative metabolism, supporting the development of memory-like CAR-T cells with enhanced persistence [9, 19, 20]. Unlike CD28-driven signaling, which favors rapid effector differentiation, 4-1BB activates TRAF-dependent pathways, including non-canonical NF-κB and p38 MAPK, leading to improved mitochondrial fitness, resistance to apoptosis, and sustained T-cell survival [19, 22]. These signaling characteristics are particularly advantageous in the nutrient-deprived and immunosuppressive tumor microenvironment of TNBC, where long-term persistence is essential for therapeutic efficacy [9, 21]. With regard to TNBC, this indicates that 4-1BB costimulation increases CAR-T cell persistence because 4-1BB costimulation supports mitochondrial health, oxidative metabolism, and resistance to apoptosis for long-term anti-tumor effects during a metabolism-exhausted state [22].

According to research on non-TNBC solid cancerous tissue models carried out in clinical research studies, CAR-T cells utilizing 4-1BB as a signal have been found to survive longer and persist longer than those utilizing CD28, but typically have slower growth (proliferation) and killing (cytotoxic) abilities initially [19, 23]. In TNBC xenograft models, 4-1BB signaling has been associated with increased CAR-T cell infiltration and sustained tumor control, highlighting its relevance for solid tumor applications [42]. However, excessive or prolonged stimulation of the 4-1BB pathway may contribute to T-cell dysfunction, indicating that an optimal balance of signaling intensity is required to maximize persistence while avoiding exhaustion [42]. In the context of triple-negative breast cancer (TNBC), HVEM-mediated costimulation may regulate CAR-T cell persistence by acting as a bidirectional signaling hub that modulates the balance between activating and inhibitory pathways. More specifically, HVEM engagement with ligands such as LIGHT supports T cell activation, survival, and function through NF-κB-dependent signaling pathways, whereas engagement of inhibitory receptors (e.g., BTLA and CD160) on T cells prevents their activation and cytokine production **(**Fig. 4**)**. Therefore, the dual signaling properties of HVEM can modulate T cell responses depending on the context in which they are stimulated, ultimately determining CAR-T cell fitness, persistence, and anti-tumor efficacy, which are determined by the relative strength of co-stimulatory and co-inhibitory signaling in the tumor microenvironment [43–45].

Recent studies have explored the integration of 4-1BB with additional costimulatory or therapeutic strategies to enhance CAR-T cell function in TNBC. For example, CAR-T cells targeting Trop-2 that incorporate multiple costimulatory domains, including 4-1BB, CD28, and CD27, have demonstrated enhanced cytotoxicity, proliferation, and cytokine production in breast cancer cell lines and three-dimensional tumor models [13]. Similarly, targeting folate receptor alpha (FRα) with 4-1BB–containing CAR-T cells has shown significant antitumor activity and improved persistence in preclinical models of breast cancer [46]. Combination approaches, such as pairing 4-1BB agonists with immune checkpoint inhibitors (e.g., PD-L1 blockade), have also demonstrated synergistic antitumor effects in TNBC models, suggesting a strategy to overcome immune suppression and enhance CAR-T cell efficacy [42].

Additional engineering strategies aim to further optimize 4-1BB signaling in CAR-T cells. Incorporation of alternative costimulatory domains, such as OX40, alongside 4-1BB has been shown to enhance cytokine production, improve T-cell function, and potentially reduce treatment-related toxicities such as cytokine release syndrome (CRS) [47]. These combinatorial designs highlight the importance of fine-tuning costimulatory signaling to balance activation, persistence, and safety in CAR-T cell therapy for solid tumors.

Despite these advances, much of the current understanding of 4-1BB signaling is derived from hematologic malignancies or non-TNBC solid tumor models, and further validation in TNBC-specific systems is required. In the context of TNBC, 4-1BB signaling is therefore more closely associated with sustained CAR-T cell persistence, although it may require optimization to achieve robust early cytotoxic responses.

Most of the data supporting the persistence-enhancing properties of 4-1BB signaling come from research on hematologic malignancies or a small number of solid tumor types [35, 48]. Studies have demonstrated that 4-1BB costimulation leads to greater mitochondrial fitness, increased aerobic metabolism, and a higher proportion of T-cells with memory T-cell phenotypes. However, it is uncertain whether these benefits will be experienced in triple-negative breast cancer (TNBC). Several studies with a TNBC focus have suggested that 4-1BB signaling can increase CAR-T cell persistence under the oxygen-poor, nutrient-deficient conditions of the TNBC tumor microenvironment, but additional studies using clinically relevant TNBC models are needed to provide definitive evidence of these effects.

### Dual costimulatory domains in cancer immunotherapy

A recent study has defined a method to enhance the effectiveness of CAR-T cells for treating breast cancer by using both CD28 and 4-1BB as costimulatory domains to create “dual costimulatory CAR-T cells.” Using both TCR activation models, the researchers demonstrated that these dual-costimulatory CAR-T cells exploit two complementary mechanisms to promote rapid T-cell proliferation and long-term persistence. Data from preclinical non-TNBC solid tumor models targeting mesothelin showed that dual costimulatory CAR-T cells were significantly more effective than conventional (non-combined) therapies in eradicating tumors and extending patient survival, and were associated with higher levels of cytokine secretion and lower levels of fatigue [47, 49]. These results suggest that dual costimulatory CAR-T cells may mitigate some of the shortcomings associated with single-costimulatory therapies (e.g., fatigue and reduced long-term persistence).

Information about co-stimulatory domains used in CAR T therapy (especially for TNBC patients) is included in Table 1. This information includes important signaling pathways, metabolic and developmental outcomes, and experimental validation systems for the co-stimulatory domains. Below are just two examples: the co-stimulatory domains (CD28 and 4-1BB) have distinct roles in regulating intracellular signaling through the PKB-AKT-mTOR and NF-κB pathways, respectively, thereby affecting T-cell metabolism (exhaustion), persistence (epigenetic regulation), and susceptibility to exhaustion (cytolytic activity) [19, 20, 24]. In solid tumors such as TNBC, these effects are further influenced by tumor microenvironmental factors, including hypoxia and immunosuppressive signaling [8, 13]. Therefore, a comprehensive comparison requires integration of construct-specific details (e.g., scFv target, CAR architecture), functional readouts (e.g., cytokine production, persistence), and model systems (e.g., in vitro, 3D cultures, xenografts). The revised table reflects these parameters, providing a more precise framework for evaluating CAR-T cell persistence in TNBC (Table 1).

**Table 1:** Mechanistic and Translational Comparison of Costimulatory Domains in CAR-T Cells (TNBC Focus).

Costimulatory Domain	Dominant Signaling Pathways	Metabolic / Phenotype Profile	Cytokine & Tonic Signaling Risk	Persistence Outcome	TNBC Model Evidence	CAR Construct (scFv / Design)	Development Stage	References
**CD28**	PI3K–AKT–mTOR	Glycolytic, effector phenotype, terminal differentiation	High cytokine production (IL-2, IFN-γ); higher tonic signaling risk	Strong early response, reduced long-term persistence	TNBC cell lines (e.g., MDA-MB-231), xenografts	HER2, MUC1, EGFR; CD28 hinge/TM commonly used	Preclinical (in vitro + in vivo)	[13, 19, 20, 24]
**4-1BB (CD137)**	TRAF → NF-κB, p38 MAPK	Oxidative metabolism, memory-like phenotype	Lower tonic signaling; sustained cytokine production	Enhanced long-term persistence	TNBC xenografts, breast cancer models	FRα, Trop-2; CD8α hinge common	Preclinical + early clinical	[22, 24, 46]
**HVEM**	NF-κB (LIGHT); inhibitory via BTLA/CD160	Context-dependent (activation vs inhibition)	Variable; may suppress function if inhibitory axis dominates	Context-dependent persistence	Limited TNBC-specific data; extrapolated from immune models	Experimental CAR constructs (varies)	Early preclinical	[33, 34, 57]
**TNFRSF9 (4-1BBL)**	NF-κB, Bcl-xL anti-apoptotic signaling	Survival-enhancing, memory-supportive	Moderate cytokine output; low apoptosis	Improved persistence (model-dependent)	Limited TNBC data; solid tumor models	Variable constructs	Preclinical	[22, 59]
**Dual (CD28** + **4-1BB)**	Combined PI3K + NF-κB signaling	Balanced effector + memory phenotype	Moderate–high cytokines; controlled tonic signaling	Improved persistence vs CD28 alone	Breast cancer models, mesothelin models	Mesothelin, Trop-2 CARs	Preclinical	[18, 24, 79]

This indicates that using two different types of co-stimulatory signals together will improve the duration CAR T-cells last by combining CD28, which triggers an immediate response from T-cells, with 4-1BB, which helps T-cells build energy stores and develop memory, allowing for both fast tumor clearance and longer-lasting results.

The structure of the CAR, along with the model used to examine it, has a major influence on how well a dual costimulation CAR functions as intended. The way that different scFv selections, spacer lengths, types of transmembrane domains, and the arrangement of costimulatory motifs affect CAR’s ability to provide effective signals, metabolic programming, and sustained activity [18, 24]. Findings from preclinical studies of solid tumors, demonstrating that dual-costimulatory signaling enhances the production of cytokines and proliferation in breast cancer, demonstrate that these enhancements are not consistently seen and are dependent on a number of factors, including the specific antigen density present, the microenvironment of the tumor, and how long the cells were exposed to an antigen [8, 18]. As such, it is important to optimize the CAR − 19 architecture based on the context of use, rather than assuming the same level of efficacy across all tumor types (e.g., TNBC).

Both the CD28 and 4-1BB costimulatory domains have had their own individual effects on CAR-T cell activity studied extensively. Each domain has both advantages and disadvantages. Cappell et al. studied CARs constructed using CD28 rather than 4-1BB. In that study, the authors reported that CD28-based CAR-T cells had a faster activation time, a quicker proliferation rate, and acute toxicity to the tumor cells compared with 4-1BB. However, these cells have an increased rate of in vivo exhaustion; therefore, they do not have as good an effect long term. 4-1BB CAR-T cells demonstrate slower T-cell proliferation; however, they do have more sustained proliferative potential over time, an improved memory profile, and higher post-treatment survival rates than CD28 CAR-T cells. The longer duration of T-cell proliferation allows for more prolonged anti-cancer activity against solid tumor types, particularly breast cancer [19]. As such, when deciding between the use of CD28 or 4-1BB as a costimulatory domain for CAR-T therapy, it is necessary to consider the type of tumor and the expected duration of response to treatment [50, 51].

### Translational implications for clinical trial design in TNBC

Different biological effects in patients with the same costimulatory domain are critical for the clinical use of CAR-T therapies in TNBC. That means designing trials for CAR-Ts is no longer just about which antigen(s) are used but also about how the costimulatory domain(s) used affects how quickly and effectively CAR-T cells expand, how long CAR-T cells persist, and the toxicity associated with those expanded and persisting CAR-T cells. CD28-based CAR-T cells appear to be particularly effective at achieving rapid tumor control in patients with bulky or rapidly growing tumors, while 4-1BB-based CAR-T therapies are likely to be most effective in providing a response for patients requiring maintenance approaches to target MRD. Evaluating CARs that utilize both costimulatory domains presents a unique opportunity to combine the advantages of both CD28 and 4-1BB costimulatory domains. Evaluating how dual-costimulatory CARs perform will require that endpoints are evaluated more thoroughly than just objective response rates, but also CAR-T persistence, metabolic fitness, and dynamics of exhaustion marker expression, as well as through longitudinal immune-monitoring studies associated with developing these types of trials [19, 47, 52].

By stratifying patients according to their tumor antigen density, their immune infiltration status, and the metabolic properties of their TNBC microenvironment, patient outcomes will be maximized. The addition of correlative studies such as single-cell transcriptomics and chromatin accessibility profiles in early phase clinical trials will be required to identify predictors of long-term response. The use of such integrated trial designs will meet the emerging regulatory requirements for gene therapy thus enabling the rapid and rational deployment of costimulative domain-optimized CAR-T cells for TNBC [10, 15, 16, 18, 21, 31].

The use of fourth-generation CAR-T cells that combine CD3ζ signaling and 4-1BB costimulation has shown promising results in breast cancer treatment, mainly targeting specific antigens like Trop2 and folate receptor alpha (FRα). The potent cytotoxicity and durable anti-cancer activity of fourth-generation CAR-T cells that targeted Trop2 is demonstrated in the context of breast cancer both in vitro (cell lines) and in vivo (PDX). In addition, adding 4-1BB costimulation enhanced the persistence and activity of Trop2-directed CAR-T cells, resulting in reduced tumor size and a lower likelihood of tumor recurrence [53]. FRα-targeting CAR-T cells showed remarkable antitumor efficacy and prolonged survival in breast cancer models [46]. These findings suggest that combining 4-1BB with the CD3ζ signaling domain improves the clinical potential of CAR-T therapies, providing a novel and potent approach to targeting breast cancer [53, 54].

Combining two Costimulatory representations in CAR-T cell Therapy has a high potential to increase the Antitumor activity of CAR-T cells in patients with cancer by enhancing both T cell Persistence and activity, which are both aspects of high activity. CD28 represents a more immediate response of the newly developed CAR-T cells; 4-1BB represents improved long-term control of tumors in most of the solid tumor types. The efficacy and long-term survival rate of the newly developed CAR-T cells in the treatment of patients with breast cancer who were either metastatic or drug-resistant has increased when both costimulatory domains, CD28 and 4-1-BB, were combined. Future research will be focused on optimizing the balance between these two costimulatory domains in order to achieve rapid and sustained responses to therapy in patients with advanced breast cancer and other solid tumors [55, 56].

## Emerging costimulatory domains: CAR-T therapy’s Hvem and Tnfrsf9

CAR-T Cell Therapy for Cancer has been the focus of many researchers, especially because it offers many benefits compared to other treatment options. The success of CAR-T in treating Leukemia and Lymphoma has spurred researchers to explore its use for solid tumors, such as TNBC. However, there are still considerable barriers to administering CAR-T to patients with one of these solid tumors, primarily arising from the many immune-inhibitory signals that the tumor microenvironment imparts to the immune system. The focus of cutting-edge research in this area is the use of novel co-stimulatory domains to enhance activating signals in T-cells and ultimately improve the efficacy of CAR-T therapies. These co-stimulatory domains include TNFRSF9 (also known as 4-1BBL), which is a member of the Tumor Necrosis Factor Receptor Superfamily, and HVEM (also known as Herpes Virus Entry Mediator). Combined with the ability to enable a T-cell to maintain its effect, co-stimulatory domains have significant potential for the patient who receives CAR-T therapy.

### HVEM as a potential domain of costimulatory control

HVEM, a tumor necrosis factor receptor superfamily component, controls immune responses. A bidirectional signaling hub that can mediate both co-stimulatory and co-inhibitory signaling based on ligand engagement, HVEM has the capacity to promote NF-kB activation and T-cell survival through binding LIGHT, while delivering inhibitory signals that inhibit T-cell activation through BTLA or CD160. In TNBC, the functionality of HVEM-mediated costimulation will probably impact through a balance of signals between two pathways (i.e., activation of LIGHT producing B-CTLA/CD160-mediated inhibition) affecting CAR-T cell durability in a manner that is highly contextual r [33, 34, 57, 58].

Although HVEM serves as a costimulatory receptor, it is a bidirectional signaling hub for various cellular functions depending on which ligands interact with it and what cell type expresses it; when it binds LIGHT it activates NF-KB and allows T lymphocytes to proliferate and survive, while when it interacts with BTLA and CD160, it provides inhibitory signals that decrease the activation of T lymphocytes and reduce the amount of cytokine that they produce [33, 57]. Importantly, opposing HVEM-based signaling pathways can coexist within the same tumor milieu, and the amount of ligand expressed determines the overall output with respect to T lymphocyte activation and effector function. In immunological and tumoral models of cancers other than TNBC, there exist studies that demonstrate that HVEM BTLA interactions inhibit T lymphocyte function, thereby promoting immune evasion, suggesting that if this pathway predominates, HVEM-based signaling could limit CAR T cell efficacy in treating TNBC [34, 58]. Therefore, this suggests that HVEM-based costimulation may not universally benefit TNBC, as it may impair T lymphocyte persistence through unmodulated inhibitory signaling. Consequently, effective incorporation into CAR T cell designs will require strategies that favor activation pathways (e.g., LIGHT-mediated pathways) and limit engagement of inhibitory ligands in the tumor milieu. The extent to which HVEM costimulation affects CAR-T cell activity is largely influenced by the type of construct used and the experimental environment. For example, the choice of antigen specificity (in this instance, HER2 and mesothelin) as well as hinge and transmembrane regions (common examples of this are those from CD8α and CD28, respectively) has been demonstrated to correlate with differences in CAR-T cell activation, persistence, and susceptibility to exhaustion in preclinical models [22, 59, 60]. Differences among tumor models—including in vitro assays, 3D cell culture, and xenograft systems—may produce functionally different results, especially in regard to solid tumors (e.g., triple-negative breast cancer), where there is antigen heterogeneity and signals to suppress the immune system [20]. Cytokine production, proliferation rate and exhaustion marker expression are other characteristics of CAR-T functionality that highlight variability with CAR-T functionality based on study [9]. Therefore, when drawing conclusions about HVEM-mediated enhancement of CAR-T function, it is critical to interpret them in light of the CAR construct’s unique design and the experimental parameters, rather than as universal effects.

Given this dual functionality, CAR designs based on HVEM will require careful engineering to bias signaling toward activation while limiting engagement of inhibitory pathways [61, 62]. Research conducted in immunological models that aren’t triple-negative breast cancer (TNBC) reveals that HVEM binds with multiple ligands: LIGHT, BTLA (B and T Lymphocyte Attenuator), and TNF-Related Weak Inducer of Apoptosis (TWEAK). Studies indicate that these ligands modulate the anti-tumor immune response and support tumor destruction [63]. Incremental developments of CAR-T therapy include an added co-stimulatory domain using HVEM; therefore, T-cell activation will increase because T-cells will be less prone to receive immunosuppressive signals from cancers. In many cases, overcoming immune evasion by cancer, a major barrier to the immune system’s response, will require the immune system to overcome it. The ligand that HVEM can bind to in the tumor microenvironment may either enhance or inhibit T-cell function. Moreover, HVEM also appears to promote the development of memory-like CAR-T phenotypes that promote long-term T cell survival and persistence [64].

### TNFRSF9 (4-1BBL) and its mechanism in T-cell proliferation and viability

TNFRSF9, also known as 4-1BBL or CD137L, has the distinct function of acting as a Costimulatory domain in CAR-T therapy. TNFRSF9 is a key regulator of T-Cell Activation, Survival and Proliferation, and due to its costimulatory properties, it can be used to promote the proliferation of CAR-T cells within the heavily depleted environment of tumors. In TNBC models, TNFRSF9 signaling may contribute to persistence by enhancing survival pathways and reducing apoptosis, although direct evidence in TNBC systems remains limited [22, 59]. In TNBCs, it is suggested that stimulation of CAR-T cytotoxic cells by TNFRSF9 promotes T-cell survival via activation of anti-apoptotic signaling cascades such as NF-κB and Bcl-xL, while enhancing metabolic fitness under long-term GPRC6A exposure.

Preclinical studies show that CAR-T cells can treat solid tumors, including TNBC, more effectively when they express TNFRSF9 [65]. In CAR-T therapy, activation of TNFRSF9 improves T-cell replication and reduces the adverse effects of T-cell fatigue, thereby lowering mortality and fatigue rates associated with CAR-T therapy. TNFRSF9 is a major contributor to the prolonged activity of CAR-T cells by preventing T-cell exhaustion. Therefore, TNFRSF9 can be used as an additional option for treating cancer types that have developed mechanisms to escape the body’s immune response through T-cell suppression and/or T-cell resistance. In addition, TNFRSF9 provides unique attributes that will also play an important role in the development of CAR-Ts for the treatment of aggressive solid tumors, such as triple-negative breast cancer (TNBC). In TNBCs, immune evasion by cancer cells significantly reduces treatment effectiveness.

### Triple-negative breast cancer: HVEM and TNFRSF9 in solid tumors

Numerous studies have indicated that the use of HVEM and TNFRSF9, along with CAR-T cells, can enhance CAR-T cell cytotoxicity against TNBC cells, as TNBC is the most aggressive subtype of breast cancer and has the fewest viable targeted therapies available for its treatment. Moreover, there are many re-emission pathways by which TNBC tumors evade immune detection. Dual co-stimulation of T cell proliferation and activation through HVEM and TNFRSF9 leads to enhanced proliferation, activation, and long-term persistence of T-cells, while simultaneously overcoming the negative signaling that affects the efficacy of CAR-T-based therapies against solid tumors by enhancing the proliferation and activation of CAR-expressing T-cells. While TNFRSF9 provides essential survival signals that help CAR-T cells resist exhaustion, HVEM enhances T-cell function by reducing immunosuppression in the tumor microenvironment [66]. These costimulatory domains, taken together, offer a strong strategy to increase the efficacy of CAR-T therapies in solid tumors, particularly those marked by immunosuppressive environments.

The introduction of new costimulatory domains, such as HVEM and TNFRSF9, into CAR-T Cell Therapy for Solid Tumors (i.e., TNBC), enhances the therapeutic potential of CAR-T Cells to treat Cancer. Both domains will work together to overcome two of the most critical obstacles in treating Solid Tumors with CAR-T cells. The determination of how to optimally incorporate TNFRSF9 and HVEM into CAR-T Cell design requires further research. The establishment of safety and efficacy in humans will occur via clinical trials, which will provide much-needed insight regarding their ability to enhance patient outcomes in solid tumors. TNFRSF9 and HVEM are likely promising candidates for next-generation CAR designs, pending validation in TNBC-specific models.

There is a lack of research on the specific function of HVEM and TNFRSF9 (4-1BB) signaling within the TNBC tumor microenvironment to inform studies that provide insight into how these pathways regulate T cell survival and activation, since most of the studies support these pathways’ involvement in T cell persistence using animal models or non-TNBC tumors [35, 67]. Additional research using TNBC-specific preclinical and clinical models will be necessary to determine whether modulation of HVEM and 4-1BB signaling is an effective mechanism to improve CAR-T cell persistence and therapeutic benefit in TNBC.

## Challenges and opportunities

### Antigen heterogeneity

Triple-negative breast cancer can undergo immune evasion as a result of the considerable antigenic diversity associated with it. Experiments are in progress on multiplexed CAR constructs targeting mesothelin, ROR1, and MUC1 to overcome tumor evasion mechanisms. The expression of antigens in triple-negative breast cancer also exhibits a unique pattern. There are two main methods to overcome the limitations of antigen diversity in triple-negative breast cancer: creating combination therapies that target multiple antigens and developing multiplex CAR constructs with costimulatory domains to enhance T-cell responses against these antigens. Ahearn et al. evaluated the impact of common genetic variants located in breast cancer risk loci on different breast cancer subtypes. They found subtype-specific genetic associations by analyzing association studies that combined information on breast cancer subtype traits with GWAS data. Several loci had increased susceptibility to ER-positive breast cancer, while other loci, including 19p13, were associated with ER-negative subtypes, indicating a genetic basis producing the heterogeneity seen in breast cancer. Additionally, various biological processes affected by functional studies demonstrate that these loci likely generate specific breast cancer subtypes by altering immune responses and hormone action. As a result, the current study demonstrates that breast cancer is a heterogeneous disease caused by several genetic factors, and suggests that further understanding of the genetics of the disease can lead to more individualized approaches to the prevention and treatment of breast cancer [68].

Examining the function of the standard ErbB-2 isoform and its nuclear variation ErbB-2 variant c, Chervo et al. found how TNBC progressed. Research on ErbB-2 variants shows that they use different yet compatible mechanisms to promote tumor development. Whereas the nuclear variant c of ErbB-2 drives transcriptional programs necessary for cancer progression, the canonical isoform primarily supports cytoplasmic signaling pathways that regulate proliferation and survival. It has been shown that the nuclear ErbB-2 variation c controls genes linked to invasion and cell cycle progression, hence increasing tumor aggressiveness. This study underscores the critical role of ErbB-2 signaling in TNBC and shows that both cytoplasmic and nuclear pathways must be targeted to effectively stop tumor progression. These results highlight the potential therapeutic benefit of developing inhibitors that could reduce the nuclear and canonical roles of ErbB-2, thereby addressing the issues posed by the aggressiveness of TNBC and the lack of targeted treatments [69].

### Persistence and exhaustion

The therapeutic efficacy of CAR-T cells is compromised by premature depletion and poor durability. The costimulatory domains that limit exhaustion paths (e.g., via IL-15 or IL-7 signaling) and enhance T-cell memory formation were explored. Investigating the co-expression of PD-1 and LAG3 on T cells in breast cancer patients, Du et al. examined how this would affect immunotherapy. LAG3 and PD-1 were co-expressed on tumor-infiltrating T cells, mostly CD8 + T cells, as determined by flow cytometry and immunohistochemistry in breast cancer tissues. With increased levels of PD-1 and LAG3, which are associated with advanced tumor stages and worse prognosis, this co-expression is linked to a fatigued T-cell phenotype characterized by diminished cytotoxic activity and reduced cytokine release. Triple-negative breast cancer (TNBC) has diverse antigen phenotypes that could allow TNBC to evade immune recognition. Research is underway to determine whether multiplexed chimeric antigen receptor (CAR) constructs can circumvent antigen heterogeneity in TNBC by activating the immune response against these antigens. Specifically, experiments are examining the development and efficacy of multiplexed CAR constructs targeting mesothelin, ROR-1, and MUC-1. The expression of antigens in TNBC is unique, exhibiting an antigenic profile distinct from other subtypes. There are two approaches to overcoming this antigenic diversity in TNBC: combinations of therapies targeting multiple antigenic epitopes and multiplex CAR constructs incorporating costimulatory domains that enhance T cell activation against these antigens. Ahearn and colleagues performed a study examining the impact of common genetic variants identified in breast cancer risk variant loci on the various breast cancer cell types. Their investigation of genetic association analyses combining breast cancer subtype characteristics with www.gwas.org identified common genetic associations among different breast cancer types, including loci associated with increased risk for ER+ breast cancer, and other loci (e.g., 19p13) that are associated with ER- subtypes, indicating that the genetic basis for heterogeneity in breast cancer is derived from these loci. Furthermore, several biological processes assessed by functional studies indicate that these loci may be the source of specific subtypes of breast cancer through their effects on immune response and hormone pathways. Therefore, the results of this analysis support the conclusion that breast cancer is a heterogeneous disease resulting from many genetic factors, and suggest that a better understanding of the genomics of breast cancer can facilitate the development of more personalized strategies for preventing and treating breast cancer [70].

This research examined current knowledge of CAR-T cell therapy for breast cancer. Furthermore, they discussed the many problems of CAR-T cell therapy for breast cancer that have arisen with recent technological advances, such as antigen heterogeneity within tumors, the immunosuppressive environment, and the activity and longevity of T cells. In addition, this discussion included recent developments in CAR-T cell engineering, such as Next-Generation CARs, which incorporate co-stimulatory domains, including ICOS and 4-1BB, to enhance T cells’ ability to be effective against tumors. In addition, they emphasized the enormous potential of bispecific CAR-T cells, as well as the combined use of CAR-T cell therapy with other immunotherapeutic strategies, including immune checkpoint inhibitors, to overcome resistance mechanisms to CAR-T treatment, thereby increasing the likelihood of a successful outcome. Additionally, this research found that aggressive subtypes of breast cancer (i.e., TNBC) may represent a viable treatment option for CAR-T cell therapy. More research is needed to optimize CAR-T cell engineering, enhance anti-tumor activity and longevity, and develop effective strategies to reduce immunosuppression associated with tumorigenesis. By utilizing multi-modal strategies that combine CAR-T cell therapy along with other forms of therapy, improvements in patient outcomes are anticipated in the future [37].

The effects of costimulatory domains on the epigenetic landscape, tolerating immunosuppressive signals, and metabolically reprogramming T cells remain active areas of study in the development of CAR T therapies for triple-negative breast cancer (TNBC). By combining ongoing studies into these areas, we can generate novel engineering solutions (i.e., combining multiple costimulatory signals and/or modifications to the metabolic programs of T cells) to overcome existing obstacles associated with CAR T therapies while also improving CAR T therapies specifically for TNBC in practical applications. The influence of costimulatory domains on T cell epigenetic states is directly related to the engineering of CAR designs. Costimulatory domains influence the transcriptional profile of CAR-T cells and, in turn, their development and use. The costimulatory domain of 4-1BB (CD137) has been shown to support the generation of a T cell phenotype strongly influenced by specific epigenetic markers associated with the stability and persistence of T cell antitumor activity [71]. To remain effective in a challenging solid-tumor environment, such as TNBC, CAR-Ts must be epigenetically reprogrammed to maintain their potency. The TNBC tumor microenvironment contains many immunosuppressive factors, such as regulatory T cells, myeloid-derived suppressor cells (MDSCs), and inhibitory cytokines like TGF-β and IL-10; therefore, the robustness of CAR-Ts to inhibition depends on which costimulatory domains are used to generate them. For example, CAR-Ts with a 4-1BB costimulatory domain have been shown to have lower expression of PD-1 and LAG-3, thus increasing the susceptibility to immune suppression due to TNBC. The ability of CAR-Ts to remain effective in the presence of these factors will ultimately determine how well they treat solid tumors [72].

T-cell activation and activity are closely linked to cellular metabolism, and the costimulatory domains on CAR-T cells dramatically influence the metabolic pathways they use. CAR-T cells expressing the CD28 costimulatory domain primarily rely on glycolytic metabolism, enabling high effector function at the expense of cell longevity and persistence. In contrast, CAR-T cells expressing the 4-1BB costimulatory domain utilize oxidative phosphorylation as the predominant metabolic pathway, thereby prolonging cell survival and maintaining lasting antitumor activity. Consequently, metabolic programming plays an important role in enabling CAR-T cells to thrive effectively within the hostile, nutrient-deprived tumor microenvironment characteristic of TNBC. The combination of two costimulatory domains, e.g., CD28 and 4-1BB, can provide synergistic benefits for CAR-T cells. The combination aims to leverage the immediate effector function mediated by CD28 and the long-term persistence associated with 4-1BB, thereby improving therapeutic benefit in TNBC. In addition, modifying CAR-T cells to express transcription factors such as FOXO1 induces a “stem-like” phenotype with enhanced mitochondrial function. The added mitochondrial capacity contributes to greater metabolic flexibility and augments CAR-T cells’ ability to accommodate the divergent metabolic demands imposed by the tumor microenvironment, ultimately increasing the anticancer effectiveness of CAR-T therapy [73].

The use of epigenetic modifiers like DNA methyltransferase inhibitors (DNMTi) and histone deacetylase inhibitors (HDACi) to alter the differentiation state of CAR-T cells increases their durability and activity. Additionally, these agents may stimulate upregulation of tumor-associated antigens in the tumor microenvironment, leading to greater vulnerability of tumor cells to CAR T-cell lysis via cytotoxic mechanisms [74].

### Safety considerations and regulatory control of costimulatory signaling

The ability of CAR-T cells to persist in the body for extended periods is important for therapeutic success; however, there are significant safety concerns associated with sustained activation of CAR-T cells via potent co-stimulatory domains. There are still significant risks associated with the use of CAR-Ts in the treatment of solid tumors that express low levels of surface targets (on-target and off-tumor toxicity) and chronic immune activation (delayed immune-related adverse events). Co-stimulatory domains, such as CD28, are also associated with an increased incidence of CRS, whereas sustained 4-1BB signaling alone may increase the risk of chronic immune activation. Therefore, it is critical to incorporate safety switches into CAR-T constructs, such as inducible suicide genes, pharmacologically controllable (on/off) systems, and tunable signaling modules [31, 75, 76].

Today, using synthetic biology technologies such as inducible promoters, synNotch circuits, and small-molecule-gated receptors, we can engineer CAR-T cells that dynamically regulate co-signaling in response to drugs or other stimuli. We have also gained the ability to regulate CAR-T cell activation, which will allow us to reduce toxicity while maintaining their effectiveness as anti-cancer therapies. For patients diagnosed with TNBC, in which antigen expression can vary widely across tumor cells, these new technologies will be highly useful. Therefore, when developing CAR-T cells for clinical use, researchers should prioritize these regulatory methods [5, 18, 22, 27].

To sum up, the type and design of costimulatory domains in CAR constructs are key factors affecting the effectiveness of CAR-T cells against TNBC (Triple Negative Breast Cancer). Understanding and altering the epigenetic (how genes are expressed) and metabolic pathways (which nutrients the cell uses) associated with these domains will lead to CAR-T cells with improved therapeutic outcomes and greater tolerance to immunosuppressive signals. Approaches such as dual costimulation, metabolic reprogramming, and epigenetic modulation are likely to eliminate most barriers to CAR-T therapy for TNBC.

### Costimulation-driven tonic signaling and persistence trade-offs

Tonic signaling, or persistent signaling of CAR-T cells in the absence of antigen, is increasingly recognized as a key factor in mediating CAR-T cell dysfunction. With respect to tonic signaling, both CD28-based CARs (which exhibit greater tonic signaling) are associated with more rapid differentiation into effector cells and an increased likelihood of exhaustion. In comparison, CARs that contain 4-1BB exhibit lower levels of tonic signaling but are less likely to induce chronic immune stimulation because they maintain low-level, persistent signaling for longer periods. These differences in tonic signaling illustrate that the effects of persistence are not simply due to increased costimulation but are also influenced by both the intensity and duration of the signals. Potential engineering strategies to reduce the negative effects of tonic signaling on CAR-T cell function include modifying the spacer region size, adjusting scFv affinity, and incorporating inducible signaling modules [35, 77].

## Future directions

Innovative costimulatory drugs targeting the TNFRSF9 (4-1BB) and HVEM receptors are expected to extend the lifespan and enhance the potency of T cells. Furthermore, research on the ongoing development of CAR-T cells has demonstrated a long-term commitment to a MEMORY-like state due to ongoing epigenetic remodeling. The emergence of combination therapies involving CAR-T treatments, immune checkpoint inhibitors, and/or oncolytic viruses may yield improved therapeutic outcomes. Numerous studies have examined the potential of ICON cells (ICOS, OX40, HVEM) to program metabolism/ to enhance persistence signature-associated properties, thereby providing an avenue for further innovation in the costimulatory arena; however, one interesting alternative is to use epigenetic modulators to restore the “stemness” of CAR-T cells and avoid exhaustion. The combination of various therapeutic strategies can counteract immunosuppression and improve durability, e.g., CAR-T cells combined with cytokine therapies, oncolytic treatments, and immune checkpoint inhibitors/antibodies. The best chance for achieving success across the continuum of care for TNBC will occur if TNBC-selective antigens (Muc1, Mesothelin, and ROR1) are well-characterized and assessed within robust preclinical model systems and well-designed clinical trials to provide forward momentum within the translational pipeline of research.

The ability of costimulatory domain signaling to influence the metabolism and epigenetic state of CAR-T cells has become evident in recent studies, showing that these three components interact closely to dictate CAR-T cell function. It has been demonstrated that different costimulatory receptors (e.g., CD28 vs. 4-1BB) regulate both immediate effector function and long-term chromatin accessibility, thereby establishing the transcriptional programs associated with memory. For instance, 4-1BB signaling creates an epigenetic landscape enriched for loci associated with stemness/memory, whereas CD28 activation yields effector-associated chromatin that is more likely to become exhausted. Developing CARs that take these metabolic and epigenetic axes into consideration, and applying additional epigenetic modulators and metabolic interventions, may provide the most effective means to preserve CAR-T cell function in the aggressive tumor microenvironment of this type of breast cancer. These synergistic efforts to combine these areas will likely lead to the development of the next generation of CAR T therapies that are durable, safe and patient-specific [19, 40–43].

Progression of CAR-T technology with customization of adaptive therapy should be advantageous for TNBC. The development of tissue-specific biomarker discovery to inform optimal costimulatory domain formulations will facilitate personalized CAR-T therapy for each TNBC patient. Cutting-edge techniques such as single-cell RNA sequencing will enable the identification and detailed characterization of tumor microenvironmental components that may enhance effectiveness at a lower cost than previous CAR-T therapies. Enhancing current approaches to CAR-T therapy by incorporating immunological profiles and tumor characteristics should maximally improve TNBC treatment outcomes.

Innovative genetic engineering methods and new cost-effective domains are being investigated to improve CAR-T cell therapy for TNBC. The current study investigates how effectively the new co-stimulatory molecules (including ICOS and 41BBL) can facilitate T-cell activation. Synthetic biology and CRISPR/Cas9 technologies enable immediate control over co-stimulatory signals, enabling greater precision in developing CAR-T cells. These innovations could provide novel strategies to address issues in the TNBC tumor microenvironment and enhance the effectiveness of CAR-T cell therapy against this aggressive form of cancer. Enhancing the co-stimulatory domains of CAR-T cells represents a new opportunity to expand the therapeutic efficacy of these therapies for TNBC, extending their duration of activity.

## Conclusion

To date, the structural elements of costimulatory signals (that is, their architecture) have been shown to represent key factors determining the persistence and therapeutic activity of CAR-T cells in patients with TNBC. The opposing biological effects of the CD28 and 4-1BB pathways imply that it is therefore important to consider how to balance the immediate cytotoxic activity associated with CD28-dependent pathways (such as the CD28/CD3 pathway) versus the longer-term cellular durability and retention associated with 4-1BB-dependent pathways (from 4-1BB/CD3 pathways). Although pathways downstream of CD28 can lead to efficient early antitumor activity, their association with a metabolically exhausted state limits the ability of CAR-T cells to generate durable (long-term) antitumor responses. Conversely, pathways downstream of 4-1BB promote the formation of a memory T cell population and persist in the body, but they may require further optimization either alone or in conjunction with other costimulatory pathways if they are to provide sufficient initial antitumor activity against solid tumors.

The addition of new and novel costimulatory domains (e.g., HVEM, TNFRSF9) can provide additional opportunities for improving the overall signaling of CAR-T cells; however, both the dual-functionality and context-dependent nature of new costimulatory domains highlight the need for them to be thoroughly studied – especially in the context of the complicated immunosuppressive environment of TNBC. Notably, increasing costimulatory signaling is not necessarily beneficial; high or uncontrolled costimulatory signaling can stimulate tonic signaling and accelerate exhaustion, increasing the risk of treatment-related toxicities. Hence, it is required that future CAR designs preferentially consider optimal modulation of the intensity, duration, and metabolism of signaling.

For the development of CAR-T therapy for TNBC, an integrated and/or multifaceted approach that balances appropriate levels of costimulatory domains and tumor-specific targeting, enhanced cellular trafficking, and the capacity to withstand the general tumor microenvironment will be essential for bringing these new and improved CARs into clinical use. The rational development of costimulatory signaling through an engineering approach grounded in mechanistic understanding and tumor-type-specific evidence will provide a viable path to overcoming current limitations and improving patient outcomes in TNBC. In TNBC, maintaining durability for CAR-T cells requires an optimization of activation level/metabolism/resistance to fatigue, rather than just increased costimulation. This review emphasizes the mechanistic interactions between co-signaling pathways and CAR T cell persistence within an emphasis on TNBC TME; therefore, this review will provide a more disease-focused perspective compared to previously published reviews. Although CAR-T cell therapy shows great promise, there are still questions about how it will perform versus other methods of treatment and how much, if any, impact it has had on patients’ quality of life while being treated for TNBC.

## Data Availability

The paper and its supplementary information contain all the data supporting this review article.
